# Multiple Risk Factors for Heart Disease: A Challenge to the Ethnopharmacological Use of *Croton urucurana* Baill.

**DOI:** 10.1155/2021/6580458

**Published:** 2021-11-15

**Authors:** Priscila Megda João Job Zago, Gustavo Ratti da Silva, Eduarda Carolina Amaral, Lorena Neris Barboza, Fernanda de Abreu Braga, Bethânia Rosa Lorençone, Aline Aparecida Macedo Marques, Karyne Garcia Tafarelo Moreno, Patrícia Regina Terço Leite, Alan de Almeida Veiga, Lauro Mera de Souza, Roosevelt Isaias Carvalho Souza, Ariany Carvalho dos Santos, João Tadeu Ribeiro-Paes, Arquimedes Gasparotto Junior, Francislaine Aparecida dos Reis Lívero

**Affiliations:** ^1^Laboratory of Preclinical Research of Natural Products, Post-Graduate Program in Medicinal Plants and Phytotherapeutics in Basic Attention, Paranaense University, Umuarama, Paraná, Brazil; ^2^Laboratory of Preclinical Research of Natural Products, Post-Graduate Program in Animal Science with Emphasis on Bioactive Products, Paranaense University, Umuarama, Paraná, Brazil; ^3^Laboratory of Cardiovascular Pharmacology, Faculty of Health Sciences, Federal University of Grande Dourados, Dourados, Mato Grosso do Sul, Brazil; ^4^Institute of Research Pelé Pequeno Príncipe, Pequeno Príncipe Faculty, Curitiba, Paraná, Brazil; ^5^Laboratory of Histology, Faculty of Health Sciences, Federal University of Grande Dourados, Dourados, Mato Grosso do Sul, Brazil; ^6^Laboratory of Genetics and Cell Therapy, São Paulo State University, Assis, São Paulo, Brazil; ^7^Laboratory of Preclinical Research of Natural Products, Post-Graduate Program in Medicinal Plants and Phytotherapeutics in Basic Attention, Post-Graduate Program in Animal Science with Emphasis on Bioactive Products, Paranaense University, Umuarama, Paraná, Brazil

## Abstract

*Croton urucurana* Baill. is a native Brazilian tree, popularly known as “sangra-d'água” or “sangue-de-dragão,” based on the red resinous sap of the trunk. Its use has been transmitted through generations based on popular tradition that attributes analgesic, anti-inflammatory, and cardioprotective properties to the tree. However, its cardioprotective effects have not yet been scientifically investigated. Thus, the present study investigated the pharmacological response to an ethanol-soluble fraction from the leaves of *C. urucurana* in Wistar rats exposed to smoking and dyslipidemia, two important cardiovascular risk factors. The extract was evaluated by high-performance liquid chromatography. Wistar rats received a 0.5% cholesterol-enriched diet and were exposed to cigarette smoke (9 cigarettes/day for 10 weeks). During the last 5 weeks, the animals were orally treated with vehicle (negative control group), *C. urucurana* extract (30, 100, and 300 mg/kg), or simvastatin (2.5 mg/kg) + enalapril (15 mg/kg). One group of rats that was not exposed to these risk factors was also evaluated (basal group). Electrocardiograms and systolic, diastolic, and mean blood pressure were measured. Blood was collected to measure total cholesterol, triglycerides, urea, and creatinine. The heart and kidneys were collected and processed for oxidative status and histopathological evaluation. The phytochemical analysis revealed different classes of flavonoids and condensed tannins. The model induced dyslipidemia and cardiac and renal oxidative stress and increased levels of urea and creatinine in the negative control group. Treatment with the *C. urucurana* extract (300 mg/kg) and simvastatin + enalapril decreased cholesterol and triglyceride levels. In contrast to simvastatin + enalapril treatment, the *C. urucurana* extract exerted cardiac and renal antioxidant effects. No alterations of electrocardiograms, blood pressure, or histopathology were observed between groups. These findings indicate that *C. urucurana* exerts lipid-lowering, renal, and cardioprotective effects against oxidative stress in a preclinical model of multiple risk factors for heart disease.

## 1. Introduction

Because of the high risk of morbidity and mortality associated with cardiovascular disease, finding ways to mitigate such risk has become paramount in public healthcare. The presence of classic risk factors, such as hypertension, dyslipidemia, obesity, sedentary lifestyle, smoking, diabetes, and family history, increases the risk of developing cardiovascular disease. Dyslipidemia is an important cardiovascular risk factor. Low-density lipoprotein cholesterol (LDL-c) is the most relevant modifiable risk factor for coronary artery disease [1]. Ample evidence indicates that low LDL-c levels are associated with a proportional reduction of cardiovascular outcomes, including myocardial infarction, stroke, and cardiovascular-related death [2].

Another cardiovascular risk factor is smoking, a disease that is caused by nicotine addiction. An estimated 1.25 billion smokers worldwide are at risk of early death from smoking [3]. The health consequences of smoking are disastrous, given long-term exposure of the body to harmful components in cigarettes. The long-term continued use of tobacco and its derivatives leads to the appearance of cardiovascular, oncological, and respiratory diseases, making it one of the main causes of preventable death worldwide [4].

Despite the high morbidity and mortality of cardiovascular disease, animal models that combine its main risk factors are scarce. Despite the existence of effective and low-cost pharmacological therapies, some new drugs that are recommended by recent guidelines are expensive or unavailable in public healthcare systems [5]. Thus, the search for new therapeutic agents that are less expensive and safe and act effectively for the management of cardiovascular risk factors is essential. Plants remain an important source of potential medicines and the development of new therapies.

One important native tree in Brazil is *Croton urucurana* Baill. (Euphorbiaceae), popularly known as “sangra-d'água” or “sangue-de-dragão.” This species is widely used by the Brazilian native population as a natural source of medicines. The leaves and bark of *C. urucurana* are popularly used to treat various conditions, including rheumatism, wounds, gastric ulcers, liver disorders, diarrhea, cancer, and cardiovascular diseases [6, 7]. The main active constituents of *C. urucurana* are tannins, lignans, and alkaloids [8]. Preclinical studies have shown that *C. urucurana* has antifungal [9], antibacterial [8], anti-inflammatory [10], antinociceptive [6, 11], antitumoral [12, 13], wound healing [12, 14], antiulcerogenic [15, 16], antidiarrheal [17, 18], and antihemorrhagic [19] effects. Toxicological studies reported that *C. urucurana* is potentially nontoxic, with an oral lethal dose 50 (LD50) above 5 g/kg in mice [9].

However, despite the popular use of *C. urucurana* for the treatment of cardiovascular diseases [7], the cardioprotective actions of this species have not yet been pharmacologically investigated. Thus, the present study investigated the lipid-lowering and antioxidant effects of an ethanol-soluble fraction obtained from leaves of *C. urucurana* in Wistar rats in a model of a combination of risk factors (exposure to tobacco smoke and dyslipidemia) for heart disease.

## 2. Material and Methods

### 2.1. Drugs

Bovine serum albumin, 5,5′-dithiobis(2-nitrobenzoic acid), reduced glutathione (GSH), xylenol orange, K_2_HPO_4_, KH_2_PO_4_, 1 M Tris, 5 mM ethylenediaminetetraacetic acid, Tris HCl (all from Sigma, St. Louis, MO, USA), pyrogallol, absolute ethanol, absolute methanol, ferrous ammonium sulfate, trichloroacetic acid, formaldehyde (all from Vetec, Rio de Janeiro, Brazil), and ultra-pure water from a Milli-Q system were used for eluent preparation.

### 2.2. Extract Preparation and Phytochemical Profile

Leaves of *Croton urucurana* Baill. were collected in May 2020 at Dourados, Mato Grosso do Sul (″22°20.9299′ south, 54°83.7713 west), and a voucher specimen (no. 5536) was deposited in the Herbarium of the Federal University of Grande Dourados. The plant was dried in an oven at 50°C for 5 days and pulverized. The extract was prepared by infusion using the methodology of Barbosa et al. [20], in which the pulverized material (100 g) was subjected to the extraction process by infusion with 1 L of boiling water. The resulting infusion was kept in an amber flask for 5 h, filtered, and then treated with 95% ethanol (1 : 3, v/v) to precipitate proteins and polysaccharides, giving rise to the heterogeneous phase that was removed by filtration. The ethanol-soluble fraction was concentrated on a rotary evaporator and lyophilized. The final yield of the dried extract of *C. urucurana* was 11.31%. Phytochemical characterization was performed using high-performance liquid chromatography (HPLC) with a diode-array detector (DAD; Shimadzu, Prominence LC-20A). Chromatography was conducted in the reverse-phase on a C18-PCP column (Ascentis Express; 150 × 4.6 mm, 2.7 *μ*m particle size) using mobile phases that were composed of (A) 0.1% formic acid in water and (B) 0.05% formic acid in acetonitrile. The separation was obtained by a gradient of B that increased from 5% to 30% in 15 min then to 80% in 20 min, with a return to 5% in 21 min and then 5 min at the initial condition for solvent reequilibration. The flow rate was 0.5 ml/min. The column temperature was held at 40°C. Compound detection was accompanied by ultraviolet (UV) light at 190–400 nm.

### 2.3. Animals

Wistar rats, weighing 150–200 g, were obtained from the central vivarium of the Federal University of Grande Dourados. The animals were housed in the vivarium of the Laboratory for Pre-Clinical Research of Natural Products, Paranaense University, with free access to food and water. The animals were housed under controlled environmental conditions (20° ± 2°C temperature, 50% ± 10% relative humidity, and 12 h/12 h light/dark cycle) with environmental enrichment. The total number of animals in the experiment was 48 (*n* = 8/group). The animals were weighed weekly on an analytical balance. The experimental protocol was approved by the Ethics Committee on the Use of Animals of Paranaense University (protocol no. 1000/2020). All national and international guidelines on animal welfare were followed. The reporting of animal investigations conformed to Animal Research Reporting of *In Vivo* Experiments (ARRIVE) guidelines [21].

### 2.4. Experimental Design

The choice of the animal species, sample size, and extract doses was based on Mendes et al. [22]. For 10 weeks, the animals received standard commercial food that was enriched with 0.5% cholesterol *ad libitum*. They were exposed to smoke from nine commercial cigarettes (0.8 mg nicotine, 10 mg tar, and 10 mg carbon monoxide) for 1 h daily, 5 days weekly, for 10 weeks, as proposed by Mendes et al. [22]. During the last 5 weeks of the experiment, the animals were treated orally by gavage with vehicle (0.1 ml of filtered water/100 g body weight; negative control [C−] group), the ethanol-soluble fraction of *Croton urucurana* (30, 100, and 300 mg/kg), or enalapril (15 mg/kg) + simvastatin (2.5 mg/kg) once daily. Nondyslipidemic and nonsmoke-exposed Wistar rats were treated with vehicle (filtered water) and served as the basal group (*n* = 8). The final groups were the following: (1) basal (rats not exposed to any risk factor and treated with vehicle), (2) negative control (C−; dyslipidemic rats exposed to cigarette smoke and treated for 5 weeks with vehicle), (3) *C. urucurana* 30 (dyslipidemic rats exposed to cigarette smoke and treated with 30 mg/kg *C. urucurana* extract for 5 weeks), (4) *C. urucurana* 100 (dyslipidemic rats exposed to cigarette smoke and treated with 100 mg/kg *C. urucurana* extract for 5 weeks), (5) *C. urucurana* 300 (dyslipidemic rats exposed to cigarette smoke and treated with 300 mg/kg *C. urucurana* extract for 5 weeks), and (6) simvastatin + enalapril (dyslipidemic rats exposed to cigarette smoke and treated with 2.5 mg/kg simvastatin plus 15 mg/kg enalapril for 5 weeks).

### 2.5. Electrocardiography and Heart Rate and Blood Pressure Measurements

On the last day of the experiment, the rats were intramuscularly anesthetized with ketamine (100 mg/kg) + xylazine (20 mg/kg). A bolus injection of heparin (15 IU) was administered subcutaneously. Electrocardiography (ECG) was recorded using a 12-lead ECG recorder (WinCardio, Micromed, Brasília, Brazil) according to Romão et al. [23]. Electrocardiographic waves were recorded for 5 min. After ECG, the left carotid artery was isolated, cannulated, and connected to a pressure transducer that was coupled to a PowerLab recording system. Chart 4.1 software (ADI Instruments, Castle Hill, Australia) was used to record heart rate, systolic blood pressure (SBP), diastolic blood pressure (DBP), and mean arterial pressure (MAP). After 15 min of stabilization, changes in heart rate and blood pressure were recorded for 5 min.

### 2.6. Blood Collection and Biochemical Analysis

Blood samples were collected from the left carotid artery using heparinized syringes. Plasma was separated by centrifugation at 1,500 × g for 10 min and stored at −80°C for biochemical analyses. Total cholesterol, triglyceride, creatinine, and urea levels were measured using commercial kits and an automated analyzer (Quick Lab).

### 2.7. Euthanasia and Organ Collection

The rats were euthanized by puncture of the diaphragm while under anesthesia. The heart and left kidney were removed, carefully dissected, and weighed on an analytical balance. The weights of the heart and kidney were multiplied by 100 and divided by the animal's body weight before euthanasia to obtain the relative organ weight (%). A sample of the heart and kidney was rapidly separated and frozen in liquid nitrogen to evaluate oxidative stress. Other organ samples were stored in a 10% formalin solution for further histological analysis.

### 2.8. Tissue Redox Status

To investigate the tissue antioxidant system, the heart and kidney samples were homogenized in a 1 : 10 dilution of potassium phosphate buffer (0.1 M, pH 6.5). Afterward, 100 *μ*l was separated, suspended in 80 *μ*l of trichloroacetic acid (12.5%), vortexed, and centrifuged at 6000 × g for 15 min at 4°C. Reduced glutathione levels were measured according to Sedlak and Lindsay [24]. The remaining homogenate was centrifuged at 9000 × g for 20 min at 4°C for the determination of superoxide dismutase (SOD) activity and lipoperoxidation (LPO) levels according to Gao et al. [25] and Jiang et al. [26], respectively.

### 2.9. Histopathological Analysis

Samples of the heart and kidney were fixed in buffered 10% formalin solution (distilled water, 35–40% formaldehyde, and monobasic and dibasic sodium phosphate), dehydrated with alcohol and xylene, embedded in paraffin, sectioned at 6 *μ*m, and stained with hematoxylin/eosin. The slides were analyzed by optical microscopy (Leica DM 2500) to evaluate cellular alterations.

### 2.10. Statistical Analysis

The data were analyzed for homogeneity of variance and a normal distribution. Differences between means were determined by one-way analysis of variance (ANOVA) followed by the Newman–Keuls *post hoc* test. The level of significance was set at 95% (*p* < 0.05). The data are expressed as the mean ± standard error of the mean (SEM).

## 3. Results

### 3.1. Phytochemical Profile

The phytochemicals in the ethanol-soluble fraction from the leaf extract of *Croton urucurana* are described in Table 1. *C. urucurana* was previously investigated for the chemical composition of its leaves and stem bark. Different compounds were reported in this plant, including flavan-3-ols, proanthocyanidins (condensed tannins), flavonols, *O*-glycosides, hydroxyflavones, *C*-glycosides [14, 27], alkaloids, terpenes, and phenolic acids [10], which were obtained with different extraction solvents. In the present study, comparisons with authentic standards identified some main compounds in the extract of the leaves of *C. urucurana*, despite the lack of information to confirm the identification of some low-abundance peaks (e.g., 1, 2, 3, and 4). Other compounds were tentatively identified based on UV spectra, supported by previous reports, but some compounds were not observed in the current extract. Alves et al. [27] performed a more comprehensive phytochemical analysis of the leaves of *C. urucurana*. They identified different classes of flavonoids, such as condensed tannins (e.g., proanthocyanidins). In the current extract, we identified peaks 5, 6, and 7 with UV spectra which were consistent with these compounds, with *λ*_max_ at 277–279 nm. Condensed tannins have been reported in all investigated parts of *C. urucurana* [10, 14, 27].

In addition to condensed tannins, Alves et al. [27] also described the presence of catechin. In the present study, comparisons with the catechin standard did not reveal catechin, but peak 9 was eluted close to catechin and had a similar UV spectrum, with *λ*_max_ at 279 nm. Although unconfirmed, the isomer epicatechin has a chromatographic elution profile that is close to catechin [28] and should be considered because it was previously reported in the bark of *C. urucurana* [10].

Flavonol-*O*-glycosides were also reported in the leaves of *C. urucurana* [27]. These compounds have characteristic UV spectra, with an absorbance *λ*_max_ range of 350–365 nm from band B and 255–265 nm from band A [29]. Compound 10 had this characteristic UV spectrum and was identified as from this class of flavonoid. In contrast, the main compound that was observed on the chromatogram was identified as rutin (peak 11, *λ*_max_ at 255 and 353 nm), confirmed by comparisons with the authentic standard. Isoquercitrin was also identified based on the authentic standard comparison at an Rt of 15.13 min, with a UV spectrum that was similar to rutin.

**Figure 1:**
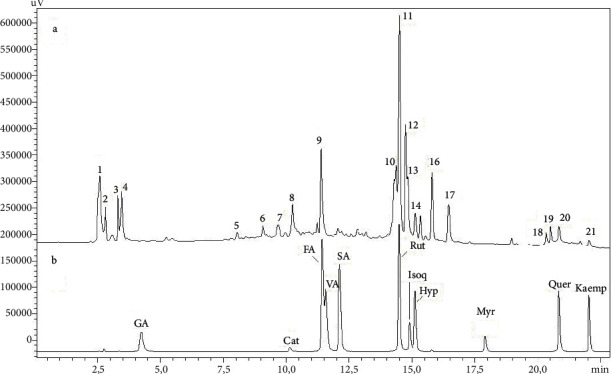
HPLC analysis of the ethanol-soluble fraction of the leaf extract of *C. urucurana* (A) and authentic standards (B). GA: gallic acid; cat: catechin; FA: ferulic acid; VA: vanillic acid; SA: syringic acid; rut: rutin; Isoq: isoquercitrin; Hyp: hyperoside; Myr: myricetin; Quer: quercetin; Kaemp: kaempferol.

Two other compounds (12 and 13) had *λ*_max_ at 269 and 337 nm, which was slightly different from the characteristic UV absorption of flavonols. Alves et al. [27] described the presence of *C*-glycosides of apigenin, and these compounds have similar UV spectra as peaks 12 and 13, suggesting they belong to this flavonoid class [30]. Peaks 15 and 16 also had characteristic UV spectra that indicated flavonoid-glycosides with *λ*_max_ at 265 and 346 nm. These compounds could not be accurately identified because in addition to rutin and isoquercitrin, Alves et al. [27] found other di- and triglycosides that were attached to myricetin, quercetin, and kaempferol. These compounds commonly appear with different glycan distributions, including several isomers, thereby hindering their identification [28, 29]. Although several glycosides were identified in the leaves of *C. urucurana*, no free aglycones were reported previously. Here, based on UV spectra and authentic standards, quercetin (peak 20) and kaempferol (peak 21) were also confirmed in the extract (Figure 1).

### 3.2. Electrocardiographic Profile, Heart Rate, and Blood Pressure

Electrocardiograms, heart rate, and blood pressure are shown in Table 2. No significant changes were found in the amplitude of the P-, Q-, R-, or S-waves or in the PR-, QRS-, QT-, and QTC-segments between experimental groups (*p* > 0.05). No significant alterations of heart rate or blood pressure were observed between groups (*p* > 0.05).

### 3.3. Body and Organ Weight

The median body weight of rats in the basal group at the end of the experiment was 166.30 ± 3.51 g. No differences were observed in body weight between groups (*p* > 0.05). For organ weights, we observed increases in relative weights (%) of the heart (*p* < 0.01) and kidney (*p* < 0.01) in the C− group compared with the basal group (0.36% ± 0.02% and 0.42% ± 0.01%, respectively). Treatment with 30, 100, and 300 mg/kg of the *C. urucurana* extract normalized relative heart weights (*p* < 0.05, 0.01, and 0.01, respectively) and relative kidney weights (*p* < 0.05, 0.01, and 0.001, respectively), whereas treatment with simvastatin + enalapril normalized only relative heart weights (*p* < 0.01). Body and organ weight are shown in Table 3.

### 3.4. Biochemical Profile

**Figure 2:**
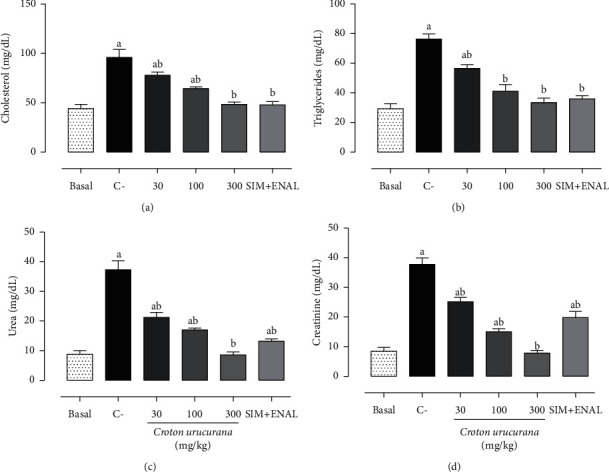
Effects of *Croton urucurana* on biochemical profile. Plasma levels of (a) cholesterol (mg/dl), (b) triglycerides (mg/dl), (c) urea (mg/dl), and (d) creatinine (mg/dl) in nondyslipidemic and nonsmoking rats (basal group) and dyslipidemic and smoking rats that were treated with vehicle (negative control (C−)), *Croton urucurana* extract (30, 100, and 300 mg/kg), or simvastatin + enalapril (SIM + ENAL). The data are expressed as mean ± SEM. ^*a*^*p* < 0.05 versus basal group; ^*b*^*p* < 0.05 versus C− group (one-way ANOVA followed by Newman–Keuls *post hoc* test).

Dyslipidemia and smoking increased plasma triglyceride and total cholesterol levels by 117.17% (*p* < 0.001) and 160.60% (*p* < 0.001), respectively, compared with the basal group. Treatment with 300 mg/kg of the *C. urucurana* extract and simvastatin + enalapril completely reversed these changes in cholesterol (*p* < 0.001) and triglycerides (*p* < 0.001), whereas treatment with 30 and 100 mg/kg of the *C. urucurana* extract only partially reversed triglyceride and total cholesterol levels. The risk factors also increased urea and creatinine levels compared with the basal group (8.83 ± 1.22 mg/dl, *p* < 0.001, and 8.50 ± 1.25 mg/dl, *p* < 0.001, respectively). Treatment with 300 mg/kg of the *C. urucurana* extract completely reversed the increases in urea (*p* < 0.001) and creatinine (*p* < 0.001) levels. Treatment with 30 and 100 mg/kg of the *C. urucurana* extract and simvastatin + enalapril partially reversed these changes (Figure 2).

### 3.5. Cardiac and Renal Redox State

**Figure 3:**
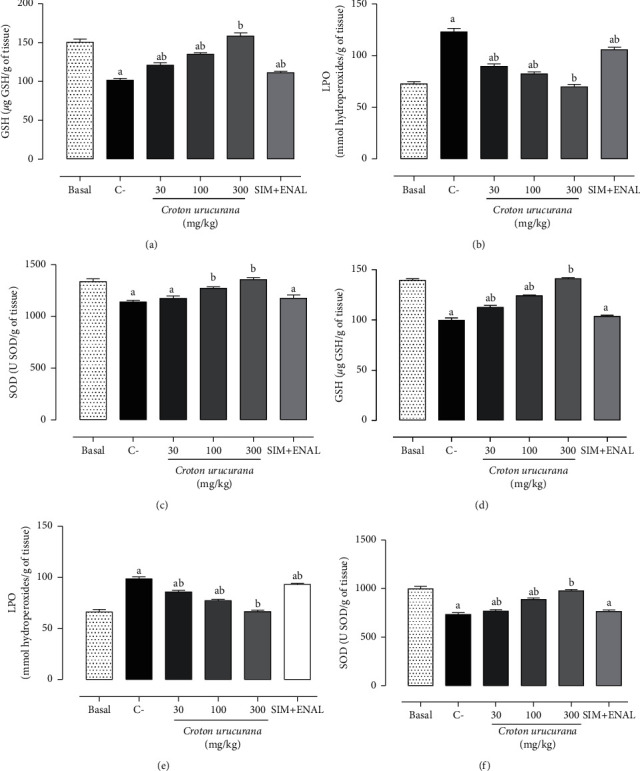
Antioxidant effects of *Croton urucurana* extract. Cardiac levels of (a) reduced glutathione, (b) lipoperoxidation, and (c) superoxide dismutase and renal levels of (d) reduced glutathione, (e) lipoperoxidation, and (f) superoxide dismutase in nondyslipidemic and nonsmoking Wistar rats (basal group) and dyslipidemic and smoking rats that were treated with vehicle (negative control (C−)), *Croton urucurana* extract (30, 100, and 300 mg/kg), or simvastatin + enalapril (SIM + ENAL). The data are expressed as mean ± SEM. ^*a*^*p* < 0.05 versus basal group; ^*b*^*p* < 0.05 versus C− group (one-way ANOVA followed by Newman–Keuls *post hoc* test).

The combination of smoking and dyslipidemia induced significant cardiac and renal oxidative stress in Wistar rats (Figure 3). Significant decreases in cardiac and renal GSH levels were observed compared with the basal group (150.50 ± 4.32 *μ*g GSH/g tissue, *p* < 0.001, and 139.30 ± 1.58 *μ*g GSH/g tissue, *p* < 0.001, respectively). These risk factors decreased cardiac (*p* < 0.001) and renal SOD (*p* < 0.001) activity and increased LPO levels compared with the basal group. Treatment with 300 mg/kg of the *C. urucurana* extract completely reversed these changes (*p* < 0.001), whereas treatment with 30 and 100 mg/kg of the *C. urucurana* extract and simvastatin + enalapril only partially reversed cardiac and renal oxidative stress.

### 3.6. Histopathological Evaluation

**Figure 4:**
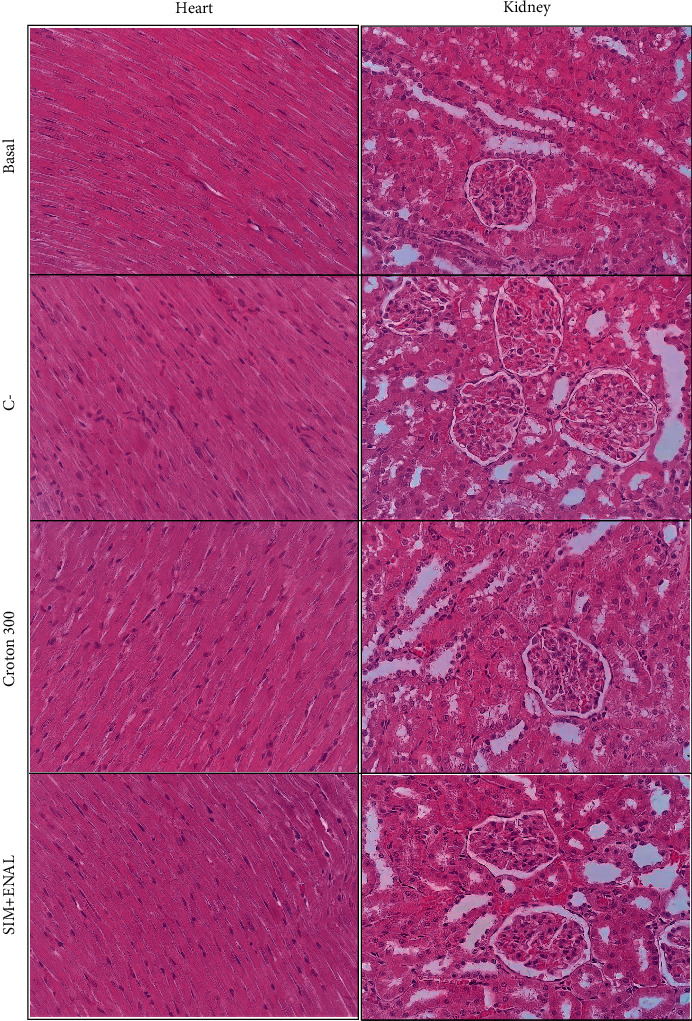
Cardiac and renal histopathological analysis. Hematoxylin/eosin staining of the heart and kidney from nondyslipidemic and nonsmoking rats (basal group) and dyslipidemic and smoking rats that were treated with vehicle (negative control (C−)), 300 mg/kg *Croton urucurana* extract (croton 300), and simvastatin + enalapril (SIM + ENAL). 40 × magnification.

Heart and kidney samples stained with hematoxylin/eosin did not reveal any significant histopathological changes in any of the experimental groups (Figure 4).

## 4. Discussion

The present study investigated cardiac and renal effects of an extract of *Croton urucurana* using an experimental model that combined dyslipidemia and tobacco smoking, both of which are important risk factors for cardiovascular diseases. Rats exposed to a high-cholesterol diet and cigarette smoke for 10 weeks exhibited dyslipidemia, cardiac and renal oxidative stress, and high plasma levels of urea and creatinine. Daily treatment with an ethanol-soluble fraction of *C. urucurana* for 5 weeks effectively reversed these changes.

Because of the high morbidity and mortality of heart diseases, finding ways to prevent them has become essential in public healthcare [31]. The presence of such risk factors as hypertension, dyslipidemia, obesity, physical inactivity, smoking, diabetes, and family history significantly increases the likelihood of heart disease. When combinations of these risk factors are present, synergistic effects can occur that further aggravate the course of cardiovascular disease [32, 33]. Despite the well-established association between multiple risk factors and heart disease, animal models that combine the main risk factors are scarce [20, 22, 33]. Thus, the use of scientifically validated preclinical models is essential to investigate and validate potential ethnobotanical candidates.

The Western medical system has a wide range of classic drugs used to prevent and control heart disease. However, their high cost and side effects limit patients' adherence to treatment. Thus, the search is ongoing for new therapeutic agents that are effective and have a more favorable side effect profile to improve treatment adherence [34]. Given the therapeutic potential of *C. urucurana* for heart disease, we performed a series of preclinical studies to validate its popular usage. The results of the present study further demonstrate the potential beneficial effects of a *C. urucurana* extract in an animal model that utilizes a combination of two well-known cardiovascular risk factors: smoking and dyslipidemia.

The HPLC-DAD analyses identified several secondary metabolites in the *C. urucurana* extract, including flavonoids (i.e., rutin) and tannins (i.e., catechin). Several studies have been conducted to determine biological effects of rutin and catechin. The preventive effects of catechins on cardiovascular disease were shown to occur through the regulation of lipid metabolism, vascular endothelial protection, and blood pressure reduction [35]. In a rat model of obesity that was induced by a high-fat diet, supplementation with rutin decreased cholesterol and triglyceride levels [36, 37]. Supplementation with rutin also exerted lipid-lowering effects (i.e., a decrease in plasma triglyceride levels) in a Golden Syrian hamster model of diet-induced hypercholesterolemia [38]. Furthermore, supplementation with rutin (100 mg/kg) improved kidney and heart morphology and function in a rat model of chronic kidney disease, promoting reno- and cardioprotective effects [39]. Antioxidant effects of rutin were also previously reported in preclinical studies [36, 40, 41]. Evidence from preclinical and clinical studies also revealed antioxidant effects of catechin [42–46]. Despite strong evidence of the participation of rutin and catechin in the lipid-lowering, antioxidant, and renoprotective effects of *C. urucurana*, we do exclude the possibility that other compounds may also be involved in its beneficial effects. We speculate that the pharmacological effects that were observed in the present study may be attributable to an integrated action of several secondary metabolites that act both independently and in a coordinated manner on different molecular targets.

The antioxidant and renoprotective effects of *C. urucurana* were superior to the simvastatin + enalapril-treated group. Although some studies showed that simvastatin exerted antioxidant effects [47, 48], we did not observe such a significant effect in the present model. Furthermore, previous studies indicated that enalapril worsened renal function in patients with heart disease by inhibiting the angiotensin-converting enzyme. Thus, enalapril appears to cause direct intrarenal vasoconstriction, reducing perfusion pressure of the renal afferent arteriole, decreasing glomerular filtration, and worsening kidney function [49–51]. Treatment with the *C. urucurana* extract did not alter renal function and instead had antioxidant actions, thus highlighting the advantage of this treatment relative to simvastatin and enalapril.

In summary, we found that treatment with the *C. urucurana* extract, mainly at the highest dose, exerted reno- and cardioprotective effects against smoking- and dyslipidemia-induced damage in Wistar rats. These results will likely contribute to further studies to validate these preclinical effects in human patients. Despite these encouraging data, the important limitations of this study are worth mentioning. First, we did not determine the molecular mechanisms by which the *C. urucurana* extract exerted its pharmacological effects. Furthermore, we did not evaluate the possible synergistic or additive effects of *C. urucurana* combined with simvastatin or enalapril. Future studies should investigate whether the cardioprotective effects of *C. urucurana* could be improved by the concomitant administration of a classic cardioprotective drug.

## 5. Conclusion

The *Croton urucurana* extract exerted reno- and cardioprotective effects in a rat model of smoking- and dyslipidemia-induced heart disease.

## Figures and Tables

**Table 1:** Phytochemicals in ethanol-soluble fraction from the leaf extract of *C. urucurana*.

Peak	*R* ^*t*^	UV *λ*_max_	Tentative identification	Reference
1	2.58	264	n.i.	—
2	2.81	200, 280	n.i.	—
3	3.31	261	n.i.	—
4	3.45	254, 278 (Sh)	n.i.	—
5	8.06	277	Condensed tannin	[27]
6	9.07	279	Condensed tannin	[27]
7	9.66	278	Condensed tannin	[27]
8	10.26	279	Condensed tannin	[27]
9	11.39	221, 268, 301	n.i.	—
10	14.36	255, 353	Flavonol-*O*-glycoside	[27]
11	14.51	255, 353	Rutin	Std. [27]
12	14.75	269, 337	Apigenin-*C*-glycoside	[27]
13	14.83	269, 337	Apigenin-*C*-glycoside	[27]
14	15.13	255, 353	Isoquercitrin	Std. [27]
15	15.33	265, 346	Flavone *C*-glycoside	[27]
16	15.81	265, 346	Flavone *C*-glycoside	[27]
17	16.46	245, 333 (Sh), 348	n.i.	—
18	20.35	229, 266, 316	n.i.	—
19	20.53	229, 266, 316	n.i.	—
20	20.87	254, 366	Quercetin	Std.
21	22.06	254, 366	Kaempferol	Std.

n.i.: not identified; Std.: compound confirmed by a comparison with authentic standard.

**Table 2:** Electrocardiogram, heart rate, and blood pressure in dyslipidemic and smoking rats that were treated with vehicle, *Croton urucurana* extract, and simvastatin + enalapril.

		Basal	C–	*Croton urucurana* (mg/kg)			SIM + ENAL
				30	100	300	
HR (BPM)		160.8 ± 5.2	152.4 ± 7.0	151.6 ± 5.6	152.3 ± 5.6	140.9 ± 20.2	171.6 ± 3.3
SBP (mm Hg)		94.0 ± 6.8	107.6 ± 11.9	100.3 ± 4.1	107.2 ± 6.2	104.6 ± 2.9	95.6 ± 11.9
DBP (mm Hg)		52.7 ± 5.2	50.4 ± 2.2	52.4 ± 3.0	58.2 ± 4.0	56.9 ± 3.7	52.6 ± 3.0
MAP (mm Hg)		73.5 ± 5.7	81.1 ± 8.8	79.1 ± 4.0	84.1 ± 5.1	82.2 ± 3.3	77.1 ± 8.9
Electrocardiogram							
Wave (mV)	P	0.07 ± 0.01	0.07 ± 0.01	0.06 ± 0.01	0.07 ± 0.01	0.07 ± 0.005	0.07 ± 0.01
	Q	−0.02 ± 0.01	−0.02 ± 0.01	−0.02 ± 0.01	−0.02 ± 0.01	−0.02 ± 0.01	−0.02 ± 0.01
	R	0.35 ± 0.01	0.34 ± 0.02	0.37 ± 0.20	0.35 ± 0.02	0.33 ± 0.02	0.31 ± 0.02
	S	−0.04 ± 0.01	−0.05 ± 0.01	−0.04 ± 0.01	−0.05 ± 0.01	−0.05 ± 0.01	−0.05 ± 0.01
						
Segment (ms)	PR	47.8 ± 3.5	43.5 ± 1.0	45.1 ± 2.7	45.5 ± 4.1	44.7 ± 4.0	43.5 ± 2.4
	QRS	42.1 ± 1.5	37.2 ± 1.7	40.3 ± 1.8	38.1 ± 2.8	37.6 ± 2.0	36.8 ± 1.6
	QT	105.8 ± 5.3	97.8 ± 3.6	96.1 ± 3.8	92.1 ± 4.4	102.3 ± 4.9	97.38 ± 5.86
	QTC	185.8 ± 7.2	183.3 ± 7.2	188.6 ± 8.4	178.4 ± 9.6	198.2 ± 7.9	187.0 ± 11.8

BPM: beats per minute; C–: negative control; DBP: diastolic blood pressure; HR: heart rate; MAP: mean arterial pressure; SBP: systolic blood pressure; SIM + ENAL: simvastatin + enalapril. *n* = 8/group. The data are expressed as mean ± SEM. One-way ANOVA followed by Newman–Keuls *post hoc* test.

**Table 3:** Body weight and relative heart and kidney weight in dyslipidemic and smoking rats that were treated with vehicle, *Croton urucurana* extract, and simvastatin + enalapril.

	Basal	C−	*Croton urucurana* (mg/kg)			SIM + ENAL
			30	100	300	
Body weight (g)	166.3 ± 3.5	176.0 ± 1.1	174.0 ± 3.1	177.7 ± 2.5	174.9 ± 3.2	175.4 ± 1.9
Heart (%)	0.36 ± 0.02	0.48 ± 0.02^a^	0.42 ± 0.01^b^	0.39 ± 0.01^b^	0.37 ± 0.01^b^	0.37 ± 0.01^b^
Kidney (%)	0.42 ± 0.01	0.49 ± 0.01^a^	0.44 ± 0.01^b^	0.42 ± 0.01^b^	0.40 ± 0.01^b^	0.46 ± 0.01

SIM + ENAL: simvastatin + enalapril. *n* = 8/group. The data are expressed as mean ± SEM. ^*a*^*p* < 0.05 versus basal; ^*b*^*p* < 0.05 versus C− (one-way ANOVA followed by Newman–Keuls *post hoc* test).

## Data Availability

Data will be available upon request to the corresponding author (e-mail: francislaine@prof.unipar.br).

## Abbreviations

ANOVA: Analysis of variance
DAD: Diode-array detector
DBP: Diastolic blood pressure
ECG: Electrocardiography
GSH: Reduced glutathione
HPLC: High-performance liquid chromatography
LD50: Lethal dose 50
LDL-c: Low-density lipoprotein cholesterol
LPO: Lipoperoxidation
MAP: Mean arterial pressure
SBP: Systolic blood pressure
SEM: Standard error of the mean
SOD: Superoxide dismutase
UV: Ultraviolet.
